# The Beneficial Role of Apigenin against Cognitive and Neurobehavioural Dysfunction: A Systematic Review of Preclinical Investigations

**DOI:** 10.3390/biomedicines12010178

**Published:** 2024-01-13

**Authors:** Tosin A. Olasehinde, Oyinlola O. Olaokun

**Affiliations:** 1 Nutrition and Toxicology Division, Food Technology Department, Federal Institute of Industrial Research Oshodi, Lagos 100261, Nigeria; 2 Department of Biology and Environmental Science, School of Science and Technology, Sefako Makgatho Health Science University, Pretoria 0204, South Africa; oyinolaokun@yahoo.com

**Keywords:** cognitive dysfunction, neurobehavioural function, neurodegenerative diseases, neuroinflammation, anxiety, depression, learning and memory, sensorimotor function, locomotion

## Abstract

Apigenin is a flavone widely present in different fruits and vegetables and has been suggested to possess neuroprotective effects against some neurological disorders. In this study, we systematically reviewed preclinical studies that investigated the effects of apigenin on learning and memory, locomotion activity, anxiety-like behaviour, depressive-like behaviour and sensorimotor and motor coordination in rats and mice with impaired memory and behaviour. We searched SCOPUS, Web of Science, PubMed and Google Scholar for relevant articles. A total of 34 studies were included in this review. The included studies revealed that apigenin enhanced learning and memory and locomotion activity, exhibited anxiolytic effects, attenuated depressive-like behaviour and improved sensorimotor and motor coordination in animals with cognitive impairment and neurobehavioural deficit. Some of the molecular and biochemical mechanisms of apigenin include activation of the ERK/CREB/BDNF signalling pathway; modulation of neurotransmitter levels and monoaminergic, cholinergic, dopaminergic and serotonergic systems; inhibition of pro-inflammatory cytokine production; and attenuation of oxidative neuronal damage. These results revealed the necessity for further research using established doses and short or long durations to ascertain effective and safe doses of apigenin. These results also point to the need for a clinical experiment to ascertain the therapeutic effect of apigenin.

## 1. Introduction

Neurological disorders are diseases that affect the nervous system, ranging from common migraine to severe brain, spine and nerve diseases [1]. Some common neurological diseases include dementia, stroke, Parkinson’s disease, lateral amyotrophic sclerosis epilepsy and multiple-system atrophy. Neurological diseases have been identified as the leading cause of mortality and disability among adults. These diseases have become a major health burden, especially among aged individuals, as they affect millions of people across the world and the cost of treatment is high [2]. The high incidence rate of neurological diseases has been linked to an increase in the age of the population, as this disease is mostly common in adults [3,4]. Apart from other pathological characteristics, some neurological disorders, especially neurodegenerative diseases, are accompanied by cognitive and neurobehavioural changes which affect quality of life [5,6]. Neuronal degeneration leads to cognitive decline and neurobehavioural impairment and has been identified in patients with AD, PD, epilepsy, stroke and MS [7]. Cognitive decline is usually characterized by impaired working memory, learning abilities, executive function and attention memory, while neurobehavioural dysfunction is commonly accompanied by anxiety, depression and loss of motor function and coordination, which are attributed to the loss and death of specific neurons such as cholinergic, dopaminergic and motor neurons in the central nervous system [8]. Several therapeutic strategies have been developed to treat the symptoms and mitigate the progression of these impairments to improve quality of life.

In the last few years, the impact of flavonoids on neurological function has been studied extensively. This class of polyphenols has shown potent neuroprotective effects against some neurodegenerative diseases in different experimental models. Apigenin is a flavone-kind of flavonoid present in fruits, teas and vegetables. It is a potent antioxidant and has been shown to exhibit anti-inflammatory, antitumorigenic and antimicrobial activities [9]. Its ability to cross the blood–brain barrier is important as it contributes to its pharmacological activity against neurological disorders [10]. Kim et al. [10] reported that apigenin exhibited a neuroprotective effect against peripheral nerve degeneration. Some studies have also established that apigenin confers antidepressant activity, which is mediated by its effect on α-adrenergic, dopaminergic and serotonergic receptors [11,12,13,14]. Apigenin improved serotonin, dopamine and epinephrine levels, which were altered in depressive animals [15,16]. Apigenin further regulates the cAMP-CREB-BDNF signalling pathway and N-methyl-D-aspartate (NMDA) receptors, which play important roles in neuronal survival, synaptic plasticity, cognitive function and mood behaviour [15]. Some experimental evidence and expert reviews have shown the pharmacological activities of apigenin, especially against some neurological diseases [17,18]. However, no systematic review has assessed the effect of apigenin on cognitive dysfunction and neurobehavioural deficit in preclinical models. This study aims to systematically review preclinical investigations that explored the cognitive-enhancing effects of apigenin and identify research gaps for further studies where needed. In this study, we explored different paradigms, including learning and memory, sensorimotor function and motor coordination, locomotion activity, anxiety-like behaviour and depressive-like behaviour, to assess the effect of apigenin on cognitive impairment and neurobehavioural deficit. We also highlighted some possible biochemical and molecular mechanisms of its therapeutic actions.

## 2. Materials and Methods

A search was conducted in four different databases (SCOPUS, Google Scholar, PubMed and Web of Science) to identify relevant studies that reported the effect of apigenin on cognitive and neurobehavioural function in preclinical experiments without limitations to regions/location or language using the Preferred Reporting Items for Systematic Reviews and Meta-Analyses (PRISMA) guidelines. The following search terms were used: “Apigenin AND (memor* OR cogniti* OR *behav* OR neuroinflammati* OR neurodegenerat* OR Alzheim* OR Parkinso*)”. Interventional studies must list the authority that provided approval and the corresponding ethical approval code.

### 2.1. Study Selection

The selection of studies for inclusion in this review was based on the following criteria: (1) that they are preclinical studies or animal studies that used mice or rats; (2) studies that used any dosage of apigenin administered for any duration versus a control group treated with stress or any chemical capable of inducing memory deficit or neurobehavioural dysfunction; and (3) that they measured outcomes that focused on memory and learning (Morris Water Maze, Barnes maze, Y-maze, T-maze, novel object recognition, passive avoidance test, inhibitory avoidance), anxiety-like behaviour (elevated plus maze, dark–light model of anxiety), depressive-like behaviour (forced swimming test, sucrose preference test, splash test, tail suspension test, sucrose splash test), sensorimotor and motor coordination (rotarod activity, grip strength, catalepsy, SG mount, sensorimotor test) and locomotion activity (open field test, locomotion activity). Articles that reported plant extracts containing apigenin were not included. Also, articles involving the use of other models such as *Drosophila melanogaster*, *Caenorhabditis elegans* and cell cultures were excluded from this study. Review articles, theses and conference papers that reported the role of apigenin on cognitive function were also excluded. Studies that reported the use of apigenin in mixtures with other compounds or bioactive constituents were also excluded. Studies that focused on the effect of apigenin on biochemical markers associated with cognitive and neurobehavioural function not indicating relevant behavioural paradigms were also excluded. Two investigators thoroughly reviewed the titles and abstracts of the articles for eligibility for inclusion in the study. Disagreement in the evaluation of eligibility for inclusion was planned to be resolved based on consensus. However, no case of disagreement emerged during the evaluation for eligibility for inclusion.

### 2.2. Data Extraction

One of the authors extracted data from the included studies while the other author checked and confirmed the extracted data. The following information was extracted from the included studies: (1) subjects; (2) dosage and route of administration; (3) duration of experiment; (4) study description; and (5) type of outcome measured. To examine the effect of apigenin using different cognitive and neurobehavioural paradigms, we categorized the measured outcome identified in the included studies into the following: learning and memory; anxiety-like behaviour, depressive-like behaviour; sensorimotor and motor coordination; and locomotion activity.

## 3. Results

### 3.1. Study Characteristics

**Figure 1:**
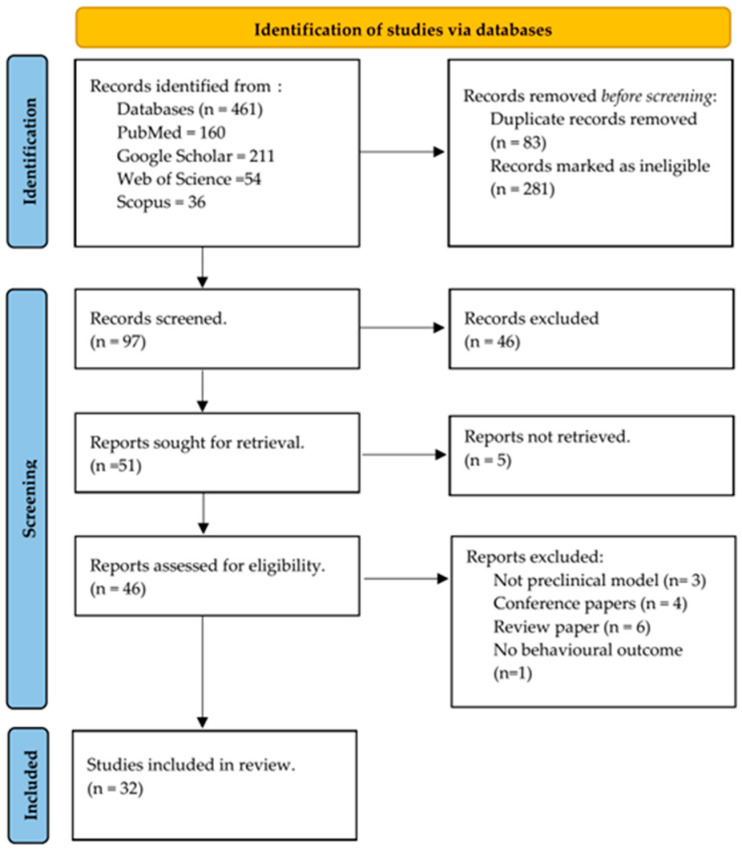
Flow diagram of article selection (based on Page et al. [19]).

As shown in Figure 1, 461 studies were identified from PubMed, Scopus, Web of Science and Google Scholar. However, 32 studies were included in the systematic review after screening with the eligibility criteria. These studies specifically address the effect of apigenin on cognitive function and behavioural outcomes in preclinical models. The hypothesis was associated with the effect of apigenin on cognitive and behavioural impairment compared to the disease group. All the included studies were performed either in mice or rats, some induced with rotenone, streptozotocin, corticosterone, chronic or mild stress, high-fat diet or fructose, lipopolysaccharide, scopolamine, methotrexate, pentylenetetrazole and acetonitrile (Table 1). The lowest dose of apigenin in the included studies was 2 mg/kg, while 351 mg/kg was the highest. Most studies used 10, 20, 40, 50 and 100 mg/kg. Also, different administration routes were used, including intragastric, intraperitoneal and oral routes, as shown in Table 1. Most of the studies used male subjects, except two studies that reported female animals. All the studies included mice or rats (Sprague Dawley or Wistar strains). The duration of the experiments in the included studies ranged from 4 days to 22 months. Twenty-two (22) cognitive and behavioural outcomes were identified in all the included studies. These include the Morris Water Maze, Barnes test, open field test, forced swimming test, splash test, rotarod test, catalepsy, elevated plus maze, Y-maze, T-maze, novel object recognition test, sucrose preference test, tail suspension test, grip strength test, passive avoidance test, locomotor activity test, inhibitory avoidance test, shuttle avoidance test, dark–light model of anxiety, pentobarbital sleeping and sensorimotor test (Table 1).

### 3.2. Learning and Memory

In the learning and memory paradigm, eight different cognitive tests were identified in the included studies (Table 2). Of the 32 included studies, only 24 reported the effect of apigenin on learning and memory. Out of these 24 studies, 12 were on the Morris Water Maze, 1 on the Barnes test, 3 studies were on the Y-maze, 2 studies on the T-maze, 2 studies reported novel recognition tests, 3 studies reported passive avoidance tests, while 1 study reported the inhibitory avoidance test. All the studies showed that apigenin improved learning and memory, except for two studies.

From the Morris Water Maze tests, eight studies showed that apigenin reduced escape latency [20,30], improved the time spent in the target quadrant [26,27,28,30], reduced first entry to the target quadrant and increased the number of crossings into the platform region [27]. Only one study reported the effect of apigenin on spatial memory using the Barnes test. The study showed that apigenin did not affect primary latency or error acquired during acquisition and did not improve spatial memory. Three studies also revealed that apigenin improved spatial working memory in animals using the Y-maze test. In these studies, apigenin restored impaired reference memory and working memory deficit by increasing the percentage of alternating behaviour in the test animals [26,27,31]. Two studies also reported that apigenin improved cognitive function using the T-maze test. In these two studies, apigenin increased the exploration of new routes and objects [29] and the percentage of spontaneous alternation [29]. Two studies that reported novel recognition tests showed that apigenin improved scopolamine-induced recognition deficit and methotrexate-induced recognition impairment.

The results from the passive avoidance test reported in two studies showed that apigenin improved retention deficit by increasing step-through latency [36] and delayed forgetting of passive avoidance response [10], while one study showed no effect on passive avoidance performance [37]. One study also revealed that apigenin did not significantly affect inhibitory avoidance activity.

### 3.3. Locomotor Activity

Two neurobehavioural paradigms were identified in the included studies, locomotor test and open field, as presented in Table 3. A total of 10 studies reported the effect of apigenin on locomotion using open field tests and locomotion tests. In the open field test, apigenin improved locomotor activity [32,35,37,42,44]. However, apigenin did not significantly affect locomotor activity in three studies, as shown by the effect on crossing [22,36], grooming and rearing [33].

### 3.4. Depressive-like Behaviour

Our findings revealed that seven studies reported the effect of apigenin on depression-like behaviour using the forced swimming test, as shown in Table 4. All the studies showed that apigenin improved immobility time in the forced swimming test. Six studies reported the use of a sucrose preference test to assess the effect of apigenin on depression-like behaviour in the intervention studies, Table 4. The results from these studies revealed that apigenin improved sucrose preference and consumption, and grooming time, and alleviated anhedonic-like behaviour in depressive mice. The tail suspension test was reported in four studies, which revealed that apigenin reduced the duration of immobility time. The splash test was also reported in one study (Table 4). The splash test revealed that apigenin improved grooming activity and locomotion in streptozotocin-induced depressive-like behaviour in a mouse model via an improvement in grooming activity.

### 3.5. Anxiety-like Behaviour Test

Of the five studies that reported the effect of apigenin on anxiety-like behaviour, four studies each reported the impact of the intervention using the elevated plus maze paradigm (Table 5). The four studies showed that apigenin attenuated anxiety-like behaviour by reducing the anxiety index and increasing the time spent in open arms and the number of entries into open arms. One study used the dark–light model of anxiety to examine the effect of apigenin on anxiety-like behaviour (Table 5). The results showed that apigenin did not exhibit an antianxiolytic effect as it did not alter the latency to the first crossing, and not did it show an effect on the time spent in the light compartment.

### 3.6. Sensorimotor Behaviour and Coordination Activity

Four studies reported the impact of apigenin on motor behaviour and performance using the rotarod test (Table 6). All the studies revealed that apigenin improved rotarod performance and enhanced muscle coordination by delaying the fall time. Two studies reported the use of the grip strength test to assess the effect of apigenin on motor coordination and muscular performance. The results showed that apigenin ameliorated the reduction in grip strength performance and improved motor coordination [23,35]. Only one study examined the effect of apigenin on motor coordination using the catalepsy test. The result from this study showed that apigenin reversed the change in cataleptic behaviour and postural instability in streptozotocin-induced depression-like behaviour in rats. Other sensorimotor function and behavioural paradigms that revealed the effect of apigenin on muscular coordination include the pole test, spontaneous activity in the cylinder and the challenging beam transversal procedure (Table 6). These tests were reported in a study by Yarim et al. [43], and the results revealed that apigenin reduced total steps but did not show any effect on step errors or error per step. Furthermore, apigenin reduced pole test scores, increased hindlimb and forelimb test scores and improved rearing and grooming in a Parkinson’s mouse model.

## 4. Discussion

To the best of our knowledge, this is the first systematic review on the effect of apigenin on cognitive and neurobehavioural outcomes in preclinical studies. Our findings revealed that apigenin exhibited cognitive-enhancing effects and improved neurobehavioural function in stress-induced animals. The studies included in this systematic review showed that apigenin improved cognitive function and neurobehaviour in impaired or stressed animals. In this study, we identified the effects of apigenin on different cognitive and neurobehavioural paradigms: learning and memory, locomotor activity, anxiety-like behaviour, depression-like behaviour and sensorimotor function.

The memory and learning paradigm revealed that apigenin exhibited nootropic effects. A significant improvement in memory and learning abilities was observed in impaired rats. Apigenin improved spatial and long-term memory, short-term and spatial working memory, recognition memory and learning abilities. However, one study reported that apigenin did not improve cognitive function in a chronic neuroinflammatory mouse model induced with glial fibrillary acidic protein-interleukin-6 (GFAP-IL-6), as revealed by the Barnes maze test [21]. The authors suggested that though other studies established that apigenin improved memory function in different models, in the context of their study, which involved chronic neuroinflammation, no improvement in cognitive function was observed.

Moreover, the study only employed the Barnes maze and did not explore other learning and memory paradigms. The molecular mechanisms of apigenin’s effect on learning and memory have been linked to synaptic transduction of the ERK/CREB/BDNF signalling pathway in the cortical cholinergic system. cAMP response element binding protein (CREB)-mediated transcription genes are essential for learning and memory. CREB also mediates the activity of glucagon-like peptide-1 (GLP-1), which in turn activates the CREB-regulated BDNF promoter [48]. Impaired ERK/CREB/BDNF signalling pathway and an inhibition of CaMK-II/PKC/PKA-ERK-CREB signalling lead to cognitive deficit and disruption of long-term potentiation in the hippocampus [49]. Furthermore, depletion of CREB and withdrawal of BDNF lead to a drastic reduction in synaptic markers in the hippocampus, which may impair long-term potentiation [50,51]. Hence, the learning and memory-enhancing effect of apigenin could be linked to its influence on CREB-BDNF signalling. Apigenin improved BDNF levels and enhanced ERK1/2 and CREB expression [21,51]. Apigenin also improved GLP-1 expression [51]. The memory-enhancing effects of apigenin were also attributed to the attenuation of oxidative stress, inhibition of apoptosis, antiamyloidegonic effects and activation of BDNF.TrkB signalling pathways [29]. Treatment with apigenin reduced Bax/Bcl-2 ratio, caspase-3 and PARP expression, hence inhibiting neuronal apoptosis and degeneration. The influence of apigenin on these signalling pathways explains the observed memory-enhancing effects.

Apigenin improved locomotion behaviour in stressed animals, as shown through three major neurobehavioural paradigms. The open field test was commonly used in all the identified studies. Two studies that reported open field tests showed that apigenin had no effect on locomotion in rats with behavioural deficits. Moreover, one of the studies revealed that apigenin did not affect locomotion. In contrast, seven other studies involving open field tests, including one that used the locomotion test, showed that apigenin improved locomotion.

Our findings also showed that apigenin markedly reduced depressive-like behaviour in preclinical studies, as shown by the results of the forced swimming test, sucrose preference test and tail suspension test from 10 included studies. Depressive-like behaviour is associated with low monoamine levels due to high monoamine oxidase activity [17,25,52]. The improvement in immobility time exhibited by apigenin in animals with depressive-like behaviour could be due to increased monoamine and serotonin levels. Hence, the mechanism of action of apigenin could be linked to the modulation of monoaminergic systems [25]. Apigenin is a potent inhibitor of monoamine oxidase [11,17]. Results from the included studies revealed apigenin as an effective natural antidepressant comparable to synthetic compounds. Some findings also attributed the antidepressant effect of apigenin to the modulation of some neurochemicals and proteins in the brain. Yi, Li, Li, Pan, Xu and Kong [16] suggested that the antidepressive effect of apigenin could be associated with multiple biochemical mechanisms, including modulation of brain monoamine, serotonin and dopamine levels; normalization of HPA axis alterations; and downregulation of the cAMP pathway. The antidepressive effect of apigenin has also been linked with its interaction with the serotonergic pathway, increasing serotonin levels and BDNF expression. Other studies also showed that apigenin exhibited antidepressive effects via increased phosphorylation of CREB and elevated levels of BDNF [38,41]. After oral treatment with apigenin, changes in the levels of serotonin and high BDNF expression were observed, and these are suggested to activate the PKA-CREB signalling pathways. Experimental investigations have also shown a link between the pathophysiology of depression and neuroinflammation. The production of pro-inflammatory cytokines contributes to the development of depression, and antidepressants have been identified as a potent inhibitor of the production of neuroinflammatory biomarkers such as cytokines. The antidepressant effect of apigenin is partly due to its anti-neuroinflammatory effects [32,33]. Apigenin inhibited cytokine production, iNos and COX-2 expression, and NF-kB activation in lipopolysaccharide depressive-like mice. Apigenin also inhibited NLRP3 inflammasome activation through the upregulation of PPAR-γ [33].

The results from the included studies showed that apigenin could be an effective alternative approach for the treatment of anxiety. There are indications that anxiety is linked to alterations in the monoaminergic system [53]. The extensive period spent in the open arms in the elevated plus maze test could be due to increased monoamine levels in the brain [54]. Amin, Ibrahim, Rizwan-ul-Hasan, Khaliq, Gabr, Muhammad, Khan, Sidhom, Tikmani, Shawky, Ahmad and Abidi [25] reported that apigenin reduced anxiety-like symptoms in rodents, and this was attributed to the modulation of brain monoamine levels and interactions with serotonin receptors. Furthermore, inflammatory processes are triggered by anxiety, which is caused by high levels of pro-inflammatory cytokines such as IL-6, IL-1β and TNF-α. This is due to the impact of these cytokines on neurotransmitters related to the anxiety response. Apigenin also inhibited proinflammatory cytokine production, which could be associated with its anxiolytic effect [11,36]. The role of oxidative stress and inflammatory-related transcription factors in the development of anxiety-like disorders has also been established [55,56]. Sharma, Sharma and Singh [38] also reported that apigenin exhibited anxiolytic effects via modulation of CREB phosphorylation and elevated BDNF levels.

**Figure 2:**
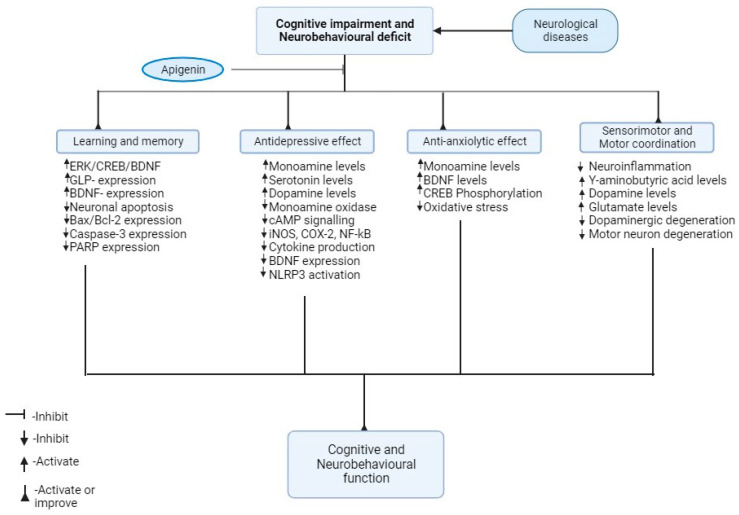
Biochemical and molecular mechanisms of apigenin against cognitive and neurobehavioural deficit.

The results from the sensorimotor tests presented in the included studies revealed that apigenin improved motor coordination and prevented loss of muscle control. In Parkinson’s disease and amyotrophic lateral sclerosis models, apigenin improved grip strength and rotarod activity, hence improving sensorimotor function [35,42,43]. Impaired motor coordination and balance in Parkinson’s disease is linked to alterations in some neurotransmitters such as dopamine, glutamate and γ-amino butyric acid (GABA) [35,57,58]. Oxidative stress and neuroinflammatory processes also contribute to motor neuron degeneration, which is important for motor coordination and sensorimotor function [35,42,43]. The identified studies showed that apigenin improved sensorimotor function and motor coordination by improving antioxidant defence and the inhibition of neuroinflammation, hence attenuating dopaminergic degeneration and, ultimately, motor neuron degeneration (Figure 2). 

## 5. Conclusions

Apigenin is a less toxic flavone widely present in many fruits and vegetables. This systematic review of preclinical trials adds to existing individual studies suggesting that apigenin can improve cognitive and neurobehavioural function. Apigenin improved learning and memory, locomotion activity, sensorimotor and motor coordination and depressive-like and anxiety-like behaviour. The learning and memory-enhancing effects of apigenin could be attributed to the modulation of BDNF levels and expression of ERL1/1 and CREB. Apigenin improved ERK/CREB/BDNF signalling which, in turn, contributed to long-term potentiation and memory function. Apigenin also attenuated hippocampal cholinergic deficit and improved acetylcholine levels, which are important for cholinergic neurotransmission and required for memory function. In addition, the antidepressive and antianxiolytic effects of apigenin are related to the inhibition of inflammatory markers (TNF-α, IL-6, IL-1β, iNOS and COX-2) and NF-kB activation; the modulation of monoamine, dopamine and serotonin levels; the normalization of HPA axis alterations; and downregulation of the cAMP pathway. Apigenin also improved sensorimotor function and motor coordination via attenuation of motor and dopaminergic degeneration; modulation of dopamine, glutamate and γ-amino butyric acid (GABA); and inhibition of ROS production and neuroinflammation.

These results show very good potential for exploring apigenin in clinical studies. Further studies can employ clinical experiments in different populations using short- and long-term trials to examine the effect of apigenin on cognitive and neurobehavioural disorders. Furthermore, different delivery systems can also be examined to establish therapeutic efficacy and minimize challenges associated with absorption, distribution and metabolism. Also, different doses of apigenin can be further explored to ascertain the safety levels around its use in treatment and its consumption through food substances.

## Figures and Tables

**Table 1:** Description of included studies.

Authors	Subjects	Dosage and Route of Administration	Duration of Experiment	Study Description	Cognitive and Behavioural Parameters Studies
Chen et al. [20]	100 male Sprague Dawley rats	50 and 100 mg/kg/intraperitoneal	7 days	Isoflurane-induced cognitive dysfunction and neuroinflammation	Morris Water Maze
Chesworth et al. [21]	Male and female (C57BL/6) and GFAP-IL6 heterozygous mice	110 mg/kg/oral	22 months	Mouse model of chronic neuroinflammation	Barnes maze
Bijani et al. [22]	Male mice of NMRI	10, 20 and 40 mg/kg intraperitoneal	4 days	Streptozotocin-induced depressive-like behaviour	Open field test Forced swimming test Splash test
Anusha and Sumathi [23]	Male Wistar rats	10 and 20 mg/kg intraperitoneal	14 days	Rotenone-induced model of Parkinson’s disease	Rotarod activity Catalepsy Rearing behaviour
Anusha et al. [24]	Male Sprague Dawley rats	10 and 20 mg/kg intraperitoneal		Rotenone-induced model of Parkinson’s disease	Rotarod test
Amin et al. [25]	Sprague Dawley rats	50 mg/kg oral	21 days	Diabetes-induced depression and anxiety	Elevated plus maze Forced swimming test
Ahmedy et al. [26]	Male Swiss Albino mice	40 mg/kg oral	7 days	Lipopolysaccharide-induced cognitive impairment in mice	Morris Water Maze Y Maze
Hashemi et al. [27]	Male Wistar rats	50 mg/kg	5 days	Kainite temporal lobe epilepsy model	Morris Water Maze Y Maze
Jameie et al. [28]	Female Wistar rats	2 mg intraperitoneal	5 weeks	Longterm ovariectomy-induced cognitive decline	Morris Water Maze
Kim et al. [29]	Male ICR mice	10 and 20 mg/kg oral	14 days	Scopolamine-induced cognitive dysfunction	T-maze Morris Water Maze Novel object recognition test
Mao et al. [30]	Male Wistar rats	10, 20 and 40 mg/kg intraperitoneal	7 weeks	Diabetes-induced cognitive deficit	Morris water maze
Nikbakht et al. [31]	Wistar rats	50 mg/kg oral	28 days	Aβ25-35-induced neurotoxicity	Y-Maze
Li et al. [32]	Male Sprague Dawley rats	20 mg/kg intragastric	3 weeks	Chronic mild stress-induced depressive behaviour	Sucrose preference test Open field test
Li et al. [33]	Male ICR mice	25 and 50 mg/kg intraperitoneal	7 days	Lipopolysaccharide-induced depressive behaviour	Tail suspension test Sucrose preference test Open field test
Liu et al. [34]	Male Kunming mice	10 and 20 mg/kg oral	8 days	Amyloid-25-35-induced toxicity in mice	Morris Water Maze
Olayinka et al. [11]	Male Mice	12.5 and 25 mg/kg intraperitoneal	14 days	Chronic stress-induced depressive-like behaviour in mice	Sucrose splash test Elevated plus maze Forced swim test Tail suspension test
Patel and Singh [35]	Male Wistar rats	25 and 50 mg P.O.	14 days	LPS-induced parkinsonism	Open field test Rotarod Grip strength test
Patil et al. [36]	Swiss Mice	5, 10 and 20 mg/kg intraperitoneal	7 dats	LPS-induced cognitive impairment	Passive avoidance test Elevated plus maze Locomotor activity Rotarod
Popovic et al. [10]	Male Wistar rats	20 mg/kg intraperitoneal	56 days	Scopolamine-induced memory impairment	Passive avoidance test
Salgueiro et al. [37]	Male Wistar rats	10 mg/kg intraperitoneal		Normal rats	Inhibitory avoidance Open field test Shuttle avoidance
Sharma et al. [38]	Swiss Albino male mice	10 and 20 mg/kg P.O.	20 days	Pentylenetetrazole-kindling-associated cognitive and behavioural impairment	T-maze Elevated plus maze Tail suspension test Forced swimming test
Taha et al. [39]	Male Sprague Dawley rats	20 mg/kg P.O.	30 days	Methotrexate-induced cognitive dysfunction	Novel object recognition Morris Water Maze
Tu et al. [40]	Male Sprague Dawley rats	20 and 40 mg/kg intraperitoneal	28 days	Post-stroke cognitive deficit in rats	Morris Water Maze
Weng et al. [41]	Male ICR mice	20 and 40 mg/kg oral	21 days	Corticosterone-induced depression-like behaviour	Sucrose preference test Forced swimming test
Yadav et al. [42]	Wistar rats	40 and 80 mg/kg P.O.		Methylmercury-induced behavioural impairment	Morris Water Maze Grip strength test Open field test Forced swim test
Yarim et al. [43]	Male C57BL/6 mice	50 mg/kg intraperitoneal	10 days	1-methyl-4-phenyl-1,2,3,6-tetrahydropyridine (MPTP)-induced Parkinson’s disease	Sensorimotor test - Challenging beam traversal procedure - Spontaneous activity in the cylinder procedure - Pole test
Yi et al. [16]	Male ICR mice and Wistar rats	10 and 20 mg/kg gastric gavage 7 and 14 mg/kg oral	2 weeks 4 weeks	Chronic mild stress-induced depressive-like behaviour	Forced swimming test Sucrose preference test
Zanoli et al. [44]	Male Sprague Dawley rats	25 mg/kg and 50 mg/kg intraperitoneal	<1 h	Behavioural characterization of apigenin	Open field test Dark-light model of anxiety Pentobarbital sleeping time
Zhang et al. [15]	C57BL/6 male mice	10, 20 and 40 mg/kg/P.O.	3 weeks	Corticosterone-induced depressive-like behaviour	Sucrose preference test Tail suspension test Open field test
Zhao [45]	Male Sprague Dawley rats	117, 234, 351 mg/kg intragastric	28 days	Acetonitrile-induced neuroinflammation in rats	Open field Test
Zhao et al. [46]	APP/PS1 double-transgenic mice and wild-type littermates	40 mg/kg oral gavage	12 weeks	AβPPswe Alzheimer’s disease mouse model	Morris Water Maze
Zhao et al. [47]	Mice	10, 20 and 40 mg/kg		Senescence-accelerated mouse prone 8 (SAMP8) mouse model	Morris Water Maze

**Table 2:** Effect of apigenin on learning and memory.

Study	Behavioural Paradigm	Results
Chen et al. [20]	Morris Water Maze	- Reduced escape latency - Improved learning and memory
Chesworth et al. [21]	Barnes maze	- High primary latency in mice (apigenin did not have any effect on primary latency) - Apigenin did not affect path length - Apigenin had no effect on error acquired during acquisition - Apigenin did not improve spatial memory in GFAP-IL6 transgenic mice
Ahmedy et al. [26]	Morris Water Maze Y-maze	- Apigenin improved time spent in the quadrant - Improved spontaneous alternation performance - Improved ability to retrieve spatial memory and form short term memories
Hashemi et al. [27]	Morris Water Maze Y-maze	- Reduced first entry to the target quadrant - Increased number of crossing into the platform region - Increased time spent in target region - Increased percentage of alternative behaviour - Restored reference memory impairment - Restored working memory deficit induced by KA
Jameie et al. [28]	Morris Water Maze	- Increased elapsed time in the target quadrant
Kim et al. [29]	Morris Water Maze Tmaze Novel object recognition	- Reduced latency to reach hidden platform - Increased time spent in the target quadrant - Increased exploration of new and old routes - Increased exploration for novel objects - Improved spatial cognitive ability - Improved scopolamine-induced recognition deficit
Mao et al. [30]	Morris Water Maze	- Reduced escape latency - Significantly reduced mean path length - Increased time spent in target quadrant
Liu et al. [34]	Morris Water Maze	- Significantly reduced escape latency - Significantly increased staying time to cross the first quadrant - Significantly increased time to cross the platform. - Improved learning and memory capabilities
Patil et al. [36]	Passive avoidance test	- Increased step-through latency
Popovic et al. [10]	Passive avoidance test	- Apigenin delayed forgetting of passive avoidance response
Salgueiro et al. [37]	Inhibitory avoidance Passive avoidance performance	- Apigenin slightly induced increase in number of crossings - No significant effect on performance of test session rearing responses - Apigenin did not show significant effect on passive avoidance performance
Sharma et al. [38]	T-maze	- Increased percentage of spontaneous alternation (T-maze)
Taha et al. [39]	Novel object recognition Morris Water Maze	- Apigenin increased discrimination index - Significantly increased preference index - Improved learning ability by reducing escape latency time
Tu et al. [40]	Morris Water Maze	- Attenuated memory acquisition deficit
Yadav et al. [42]	Morris Water Maze	- Restored long-term memory deficit via reduction in escape latency and increase in time spent in target quadrant
Zhao et al. [46]	Morris Water Maze	- Exhibited significant effect on escape latency and recovered spatial learning deficit - In the probe trail, apigenin increased time spent in the target quadrant - Increased number of crossings, thereby improving spatial memory capability
Zhao et al. [47]	Morris Water Maze	- Reduced escape latency - Increased time spent in target quadrant and number of crossings of the platform
Nikbakht et al. [31]	Y-maze	- Improved spatial working memory

**Table 3:** Effect of apigenin on locomotor behaviour.

Study	Behavioural Paradigm	Results
Bijani et al. [22]	Open field test	- Apigenin showed no effect on the alteration in locomotion
Li et al. [32]	Open field test	- Improved locomotor activity
Li et al. [33]	Open field test	- Apigenin did not show significant effect on crossing, grooming or rearing
Patel and Singh [35]	Open field test	- Increased locomotor activity
Patil et al. [36]	Locomotor activity	- Apigenin did not show any effect on locomotor activity.
Salgueiro et al. [37]	Open field test	- No significant effect on performance of test session rearing responses
Yadav et al. [42]	Open field test	- Improved locomotion and rearing activity
Zanoli et al. [44]	Open field test	- Reduced number of crossings and rearings - Reduced locomotor behaviour
Zhang et al. [15]	Open field test	- Reduced immobility time - Increased total moving distance and reduced immobility time
Zhao et al. [45]	Open field test	- Reduced total distance of motion via reduction in autonomic activity

**Table 4:** Effect of apigenin on depressive-like behaviour.

Study	Behavioural Paradigm	Results
Bijani et al. [22]	Forced swimming test Splash test	- Apigenin improved immobility time in FST - Apigenin improved grooming activity in splash test
Amin et al. [25]	Forced swimming test	- Improved mobility time in forced swimming test
Li et al. [32]	Sucrose preference test	- Improved sucrose consumption and prevented elicited anhedonia and antidepressant-like symptoms
Li et al. [33]	Tail suspension test Sucrose preference test	- Reduced immobility duration - Increased percentage sucrose consumption
Olayinka et al. [11]	Sucrose splash test Forced swim test Tail suspension test	- Significantly increased duration of grooming - Alleviated anhedonic-like behaviour in depressive mice via increase in grooming time - Reduced the duration of immobility
Sharma et al. [38]	Tail suspension test Forced swimming test	- Significant reduction in the duration of immobility time
Weng et al. [41]	Sucrose preference test Forced swimming test	- Apigenin improved sucrose preference in mice - Reduced immobility time in mice
Yadav et al. [42]	Forced swim test	- Markedly reduced immobility time
Yi et al. [16]	Forced swimming test Sucrose preference test	- Apigenin at 10 and 20 mg/kg reduced immobility time - Attenuated CMS-induced deficit in sucrose intake by increasing levels of sucrose consumption
Zhang et al. [15]	Sucrose preference test Tail suspension test	- Significantly reversed reduction in sucrose consumption in rats - Reduced immobility time

**Table 5:** Effect of apigenin on anxiety-like behaviours.

Study	Behavioural Paradigm	Results
Amin et al. [25]	Elevated plus maze	- Increased time spent in open arms, and also increased number entries into open arms
Olayinka et al. [11]	Elevated plus maze	- Increased frequency of open arm entry - Increased duration of mice in the open arm
Patil et al. [36]	Elevated plus maze	- Decreased transfer latency (EPM)
Sharma et al. [38]	Elevated plus maze	- Markedly reduced anxiety index
Zanoli et al. [44]	Dark–light model of anxiety	- Did not alter latency to the first crossing nor time spent in the light compartment.

**Table 6:** Effect of apigenin on sensorimotor function and motor coordination.

Study	Behavioural Paradigm	Results
Anusha and Sumathi [23]	Rotarod activity Grip strength Catalepsy	- Apigenin improved muscle grip strength but did not produce a significant effect on grip strength performance - Reversed change in cataleptic behaviour - Reversed postural instability
Anusha et al. [24]	Rotarod	- Apigenin improved muscle coordination by delaying fall time Improved grip strength performance
Patel and Singh [35]	Rotarod Grip strength test SG mount	- Ameliorated reduction in rotarod activity - Ameliorated reduction in grip strength performance (motor coordination) - Significantly attenuated increased climbing and get off time
Patil et al. [36]	Rotarod	- Apigenin improved rotarod performance in aged mice at higher doses (20 mg); lower doses did not show any effect
Yadav et al. [42]	Grip strength test	- Improved grip strength force
Yarim et al. [43]	Sensorimotor test - Challenging beam traversal procedure - Spontaneous activity in the cylinder procedure - Pole test	- Apigenin reduced total steps - Did not show any effect on step errors or errors per step - Reduced forelimb steps - Increased forelimb and hindlimb steps - Increased rearing number and grooming time

## Data Availability

Not applicable.

## References

[1] Dumurgier J., Tzourio C. (2020). Epidemiology of neurological diseases in older adults. Rev. Neurol..

[2] Van Schependom J., D’Haeseleer M. (2023). Advances in Neurodegenerative Diseases. J. Clin. Med..

[3] Morris J.C. (2013). Neurodegenerative disorders of aging: The down side of rising longevity. Mo. Med..

[4] Gómez-Gómez M.E., Zapico S.C. (2019). Frailty, Cognitive Decline, Neurodegenerative Diseases and Nutrition Interventions. Int. J. Mol. Sci..

[5] Levenson R.W., Sturm V.E., Haase C.M. (2014). Emotional and behavioral symptoms in neurodegenerative disease: A model for studying the neural bases of psychopathology. Annu. Rev. Clin. Psychol..

[6] Ayeni E.A., Aldossary A.M., Ayejoto D.A., Gbadegesin L.A., Alshehri A.A., Alfassam H.A., Afewerky H.K., Almughem F.A., Bello S.M., Tawfik E.A. (2022). Neurodegenerative Diseases: Implications of Environmental and Climatic Influences on Neurotransmitters and Neuronal Hormones Activities. Int. J. Environ. Res. Public Health.

[7] Migliaccio R., Tanguy D., Bouzigues A., Sezer I., Dubois B., Le Ber I., Batrancourt B., Godefroy V., Levy R. (2020). Cognitive and behavioural inhibition deficits in neurodegenerative dementias. Cortex.

[8] Calina D., Buga A.M., Mitroi M., Buha A., Caruntu C., Scheau C., Bouyahya A., El Omari N., El Menyiy N., Docea A.O. (2020). The Treatment of Cognitive, Behavioural and Motor Impairments from Brain Injury and Neurodegenerative Diseases through Cannabinoid System Modulation-Evidence from In Vivo Studies. J. Clin. Med..

[9] Cirmi S., Ferlazzo N., Lombardo G.E., Ventura-Spagnolo E., Gangemi S., Calapai G., Navarra M. (2016). Neurodegenerative Diseases: Might *Citrus* Flavonoids Play a Protective Role?. Molecules.

[10] Popović M., Caballero-Bleda M., Benavente-García O., Castillo J. (2014). The flavonoid apigenin delays forgetting of passive avoidance conditioning in rats. J. Psychopharmacol..

[11] Olayinka J.N., Akawa O.B., Ogbu E.K., Eduviere A.T., Ozolua R.I., Soliman M. (2023). Apigenin attenuates depressive-like behavior via modulating monoamine oxidase A enzyme activity in chronically stressed mice. Curr. Res. Pharmacol. Drug Discov..

[12] Al-Yamani M.J., Mohammed Basheeruddin Asdaq S., Alamri A.S., Alsanie W.F., Alhomrani M., Alsalman A.J., Al Mohaini M., Al Hawaj M.A., Alanazi A.A., Alanzi K.D. (2022). The role of serotonergic and catecholaminergic systems for possible antidepressant activity of apigenin. Saudi J. Biol. Sci..

[13] Zhang X., Bu H., Jiang Y., Sun G., Jiang R., Huang X., Duan H., Huang Z., Wu Q. (2019). The antidepressant effects of apigenin are associated with the promotion of autophagy via the mTOR/AMPK/ULK1 pathway. Mol. Med. Rep..

[14] Alghamdi A., Almuqbil M., Alrofaidi M.A., Burzangi A.S., Alshamrani A.A., Alzahrani A.R., Kamal M., Imran M., Alshehri S., Mannasaheb B.A. (2022). Potential Antioxidant Activity of Apigenin in the Obviating Stress-Mediated Depressive Symptoms of Experimental Mice. Molecules.

[15] Zhang L., Lu R.R., Xu R.H., Wang H.H., Feng W.S., Zheng X.K. (2023). Naringenin and apigenin ameliorates corticosterone-induced depressive behaviors. Heliyon.

[16] Yi L.T., Li H.M., Li Y.C., Pan Y., Xu Q., Kong L.D. (2008). Antidepressant-like behavioral and neurochemical effects of the citrus-associated chemical apigenin. Life Sci..

[17] Nabavi S.F., Khan H., D’Onofrio G., Šamec D., Shirooie S., Dehpour A.R., Argüelles S., Habtemariam S., Sobarzo-Sanchez E. (2018). Apigenin as neuroprotective agent: Of mice and men. Pharmacol. Res..

[18] Gaur K., Siddique Y.H. (2023). Effect of apigenin on neurodegenerative diseases. CNS & Neurological Disorders-Drug Targets.

[19] Page M.J., McKenzie J.E., Bossuyt P.M., Boutron I., Hoffmann T.C., Mulrow C.D., Shamseer L., Tetzlaff J.M., Akl E.A., Brennan S.E. (2021). The PRISMA 2020 statement: An updated guideline for reporting systematic reviews. Syst. Rev..

[20] Chen L., Xie W., Xie W., Zhuang W., Jiang C., Liu N. (2017). Apigenin attenuates isoflurane-induced cognitive dysfunction via epigenetic regulation and neuroinflammation in aged rats. Arch. Gerontol. Geriatr..

[21] Chesworth R., Gamage R., Ullah F., Sonego S., Millington C., Fernandez A., Liang H., Karl T., Münch G., Niedermayer G. (2021). Spatial memory and microglia activation in a mouse model of chronic neuroinflammation and the anti-inflammatory effects of apigenin. Front. Neurosci..

[22] Bijani S., Dizaji R., Sharafi A., Hosseini M.J. (2022). Neuroprotective Effect of Apigenin on Depressive-Like Behavior: Mechanistic Approach. Neurochem. Res..

[23] Anusha C., Sumathi T. (2016). Protective role of apigenin against rotenone induced model of parkinson’s disease: Behavioral study. Int. J. Toxicol. Pharmacol. Res..

[24] Anusha C., Sumathi T., Joseph L.D. (2017). Protective role of apigenin on rotenone induced rat model of Parkinson’s disease: Suppression of neuroinflammation and oxidative stress mediated apoptosis. Chem.-Biol. Interact..

[25] Amin F., Ibrahim M.A.A., Rizwan-ul-Hasan S., Khaliq S., Gabr G.A., Muhammad, Khan A., Sidhom P.A., Tikmani P., Shawky A.M. (2022). Interactions of Apigenin and Safranal with the 5HT1A and 5HT2A Receptors and Behavioral Effects in Depression and Anxiety: A Molecular Docking, Lipid-Mediated Molecular Dynamics, and In Vivo Analysis. Molecules.

[26] Ahmedy O.A., Abdelghany T.M., El-Shamarka M.E.A., Khattab M.A., El-Tanbouly D.M. (2022). Apigenin attenuates LPS-induced neurotoxicity and cognitive impairment in mice via promoting mitochondrial fusion/mitophagy: Role of SIRT3/PINK1/Parkin pathway. Psychopharmacology.

[27] Hashemi P., Fahanik Babaei J., Vazifekhah S., Nikbakht F. (2019). Evaluation of the neuroprotective, anticonvulsant, and cognition-improvement effects of apigenin in temporal lobe epilepsy: Involvement of the mitochondrial apoptotic pathway. Iran. J. Basic Med. Sci..

[28] Jameie S.B., Pirasteh A., Naseri A., Jameie M.S., Farhadi M., Babaee J.F., Elyasi L. (2021). β-Amyloid Formation, Memory, and Learning Decline Following Long-term Ovariectomy and Its Inhibition by Systemic Administration of Apigenin and β-Estradiol. Basic Clin. Neurosci..

[29] Kim Y., Kim J., He M., Lee A., Cho E. (2021). Apigenin Ameliorates Scopolamine-Induced Cognitive Dysfunction and Neuronal Damage in Mice. Molecules.

[30] Mao X.Y., Yu J., Liu Z.Q., Zhou H.H. (2015). Apigenin attenuates diabetes-associated cognitive decline in rats via suppressing oxidative stress and nitric oxide synthase pathway. Int. J. Clin. Exp. Med..

[31] Nikbakht F., Khadem Y., Haghani S., Hoseininia H., Moein Sadat A., Heshemi P., Jamali N. (2019). Protective Role of Apigenin Against Aβ 25-35 Toxicity Via Inhibition of Mitochondrial Cytochrome c Release. Basic Clin. Neurosci..

[32] Li R., Wang X., Qin T., Qu R., Ma S. (2016). Apigenin ameliorates chronic mild stress-induced depressive behavior by inhibiting interleukin-1β production and NLRP3 inflammasome activation in the rat brain. Behav. Brain Res..

[33] Li R., Zhao D., Qu R., Fu Q., Ma S. (2015). The effects of apigenin on lipopolysaccharide-induced depressive-like behavior in mice. Neurosci. Lett..

[34] Liu R., Zhang T., Yang H., Lan X., Ying J., Du G. (2011). The flavonoid apigenin protects brain neurovascular coupling against amyloid-β_25–35_-induced toxicity in mice. J. Alzheimers Dis..

[35] Patel M., Singh S. (2022). Apigenin Attenuates Functional and Structural Alterations via Targeting NF-kB/Nrf2 Signaling Pathway in LPS-Induced Parkinsonism in Experimental Rats: Apigenin Attenuates LPS-Induced Parkinsonism in Experimental Rats. Neurotox. Res..

[36] Patil C.S., Singh V.P., Satyanarayan P.S., Jain N.K., Singh A., Kulkarni S.K. (2003). Protective effect of flavonoids against aging- and lipopolysaccharide-induced cognitive impairment in mice. Pharmacology.

[37] Salgueiro J.B., Ardenghi P., Dias M., Ferreira M.B., Izquierdo I., Medina J.H. (1997). Anxiolytic natural and synthetic flavonoid ligands of the central benzodiazepine receptor have no effect on memory tasks in rats. Pharmacol. Biochem. Behav..

[38] Sharma P., Sharma S., Singh D. (2020). Apigenin reverses behavioural impairments and cognitive decline in kindled mice via CREB-BDNF upregulation in the hippocampus. Nutr. Neurosci..

[39] Taha M., Eldemerdash O.M., Elshaffei I.M., Yousef E.M., Soliman A.S., Senousy M.A. (2023). Apigenin Attenuates Hippocampal Microglial Activation and Restores Cognitive Function in Methotrexate-Treated Rats: Targeting the miR-15a/ROCK-1/ERK1/2 Pathway. Mol. Neurobiol..

[40] Tu F., Pang Q., Huang T., Zhao Y., Liu M., Chen X. (2017). Apigenin ameliorates post-stroke cognitive deficits in rats through histone acetylation- mediated neurochemical alterations. Med. Sci. Monit..

[41] Weng L., Guo X., Li Y., Yang X., Han Y. (2016). Apigenin reverses depression-like behavior induced by chronic corticosterone treatment in mice. Eur. J. Pharmacol..

[42] Yadav R.K., Mehan S., Sahu R., Kumar S., Khan A., Makeen H.A., Al Bratty M. (2022). Protective effects of apigenin on methylmercury-induced behavioral/neurochemical abnormalities and neurotoxicity in rats. Hum. Exp. Toxicol..

[43] Yarim G.F., Kazak F., Yarim M., Sozmen M., Genc B., Ertekin A., Gokceoglu A. (2022). Apigenin alleviates neuroinflammation in a mouse model of Parkinson’s disease. Int. J. Neurosci..

[44] Zanoli P., Avallone R., Baraldi M. (2000). Behavioral characterisation of the flavonoids apigenin and chrysin. Fitoterapia.

[45] Zhao F., Dang Y., Zhang R., Jing G., Liang W., Xie L., Li Z. (2019). Apigenin attenuates acrylonitrile-induced neuro-inflammation in rats: Involved of inactivation of the TLR4/NF-κB signaling pathway. Int. Immunopharmacol..

[46] Zhao L., Wang J.-L., Liu R., Li X.-X., Li J.-F., Zhang L. (2013). Neuroprotective, anti-amyloidogenic and neurotrophic effects of apigenin in an Alzheimer’s disease mouse model. Molecules.

[47] Zhao Y., Li F., Zeng Y. (2015). Effects of apigenin on learning and memory and synaptic plicity in SAMP8 mice. Chin. J. Gerontol..

[48] Bourtchuladze R., Frenguelli B., Blendy J., Cioffi D., Schutz G., Silva A.J. (1994). Deficient long-term memory in mice with a targeted mutation of the cAMP-responsive element-binding protein. Cell.

[49] Lyu Y., Ren X.K., Zhang H.F., Tian F.J., Mu J.B., Zheng J.P. (2020). Sub-chronic administration of benzo[a]pyrene disrupts hippocampal long-term potentiation via inhibiting CaMK II/PKC/PKA-ERK-CREB signaling in rats. Environ. Toxicol..

[50] Velmurugan K., Balamurugan A.N., Loganathan G., Ahmad A., Hering B.J., Pugazhenthi S. (2012). Antiapoptotic actions of exendin-4 against hypoxia and cytokines are augmented by CREB. Endocrinology.

[51] Kalivarathan J., Chandrasekaran S.P., Kalaivanan K., Ramachandran V., Carani Venkatraman A. (2017). Apigenin attenuates hippocampal oxidative events, inflammation and pathological alterations in rats fed high fat, fructose diet. Biomed. Pharmacother..

[52] Miura H., Naoi M., Nakahara D., Ohta T., Nagatsu T. (1993). Changes in monoamine levels in mouse brain elicited by forced-swimming stress, and the protective effect of a new monoamine oxidase inhibitor, RS-8359. J. Neural Transm..

[53] Liu Y., Zhao J., Guo W. (2018). Emotional Roles of Mono-Aminergic Neurotransmitters in Major Depressive Disorder and Anxiety Disorders. Front. Psychol..

[54] Cryan J.F., Page M.E., Lucki I. (2005). Differential behavioral effects of the antidepressants reboxetine, fluoxetine, and moclobemide in a modified forced swim test following chronic treatment. Psychopharmacology.

[55] Salim S., Chugh G., Asghar M., Donev R. (2012). Inflammation in anxiety. Advances in Protein Chemistry and Structural Biology.

[56] Quesseveur G., David D.J., Gaillard M.C., Pla P., Wu M.V., Nguyen H.T., Nicolas V., Auregan G., David I., Dranovsky A. (2013). BDNF overexpression in mouse hippocampal astrocytes promotes local neurogenesis and elicits anxiolytic-like activities. Transl. Psychiatry.

[57] Moore A.H., Bigbee M.J., Boynton G.E., Wakeham C.M., Rosenheim H.M., Staral C.J., Morrissey J.L., Hund A.K. (2010). Non-Steroidal Anti-Inflammatory Drugs in Alzheimer’s Disease and Parkinson’s Disease: Reconsidering the Role of Neuroinflammation. Pharmaceuticals.

[58] Gardoni F., Bellone C. (2015). Modulation of the glutamatergic transmission by Dopamine: A focus on Parkinson, Huntington and Addiction diseases. Front. Cell. Neurosci..

